# Scoliosis in a Patient With Gilbert Syndrome: A Case Report and Review of the Literature

**DOI:** 10.1097/MD.0000000000002147

**Published:** 2015-10-30

**Authors:** Zheng Li, Jianxiong Shen, Jinqian Liang

**Affiliations:** Department of Orthopaedic Surgery, Peking Union Medical College Hospital, Chinese Academy of Medical Sciences and Peking Union Medical College, Beijing, China.

## Abstract

Gilbert syndrome (GS) is mainly characterized by intermittent unconjugated hyperbilirubinemia in the absence of hepatocellular disease or hemolysis. Little data are available on operative outcomes in GS patients with spinal deformity surgery.

This study has presented a case of GS occurring in the patient with scoliosis.

The patient was a 30-year-old female with scoliosis and GS. She was taken a correction form Thoracic 2 to Lumbar 1) levels by using the USS-II spinal system. At 2 years follow-up, the patient was well balanced and pain free. Plain radiographs demonstrated spine solid fusion without correction loss.

Although complex scoliosis surgery can be performed safely in these patients with GS, careful perioperative managements including liver function and coagulation function are required.

## INTRODUCTION

Gilbert Syndrome (GS) is a common autosomal dominant hereditary condition characterized by recurrent mild unconjugated hyperbilirubinaemia in the absence of haemolysis or underlying liver diseases.^1,2^ Augustin Gilbert and Pierre Lereboullet first reported this disease in 1901. GS is rarely diagnosed before puberty though it is a congenital disease.^1,3^ One explanation is hormonal changes of puberty. The syndrome is found in 7% of general population, and it is more common among men than women with the ratio of 2–7:1.^4,5^ The typical presentation of this syndrome is mild unconjugated hyperbilirubinaemia, and indirect bilirubin levels usually exceed the upper limit of normal after fasting, dehydration, menstruation, intercurrent disease, and overexertion.^6,7^ Other nonspecific symptoms including epigastric fullness, abdominal pain, fat intolerance, and fatigue have also been reported.^8,9^ Fusion surgery of spinal deformity with hyperbilirubinemia may induce liver dysfunction or multiple organ failure. Preoperative hyperbilirubinemia level is one of the risk factors for operative mortality. However, the relation between operative risks of fusion surgery of spinal deformity and hyperbilirubinemia remains unknown. We here present a case of GS in a 30-year-old girl with unusual presentation: scoliosis.

## CONSENT

Written informed consent was obtained from the parents on behalf of the child for publication of this case report and any accompanying images. A copy of the written consent is available for review by the Editor of this journal.

## CASE REPORT

**FIGURE 1:**
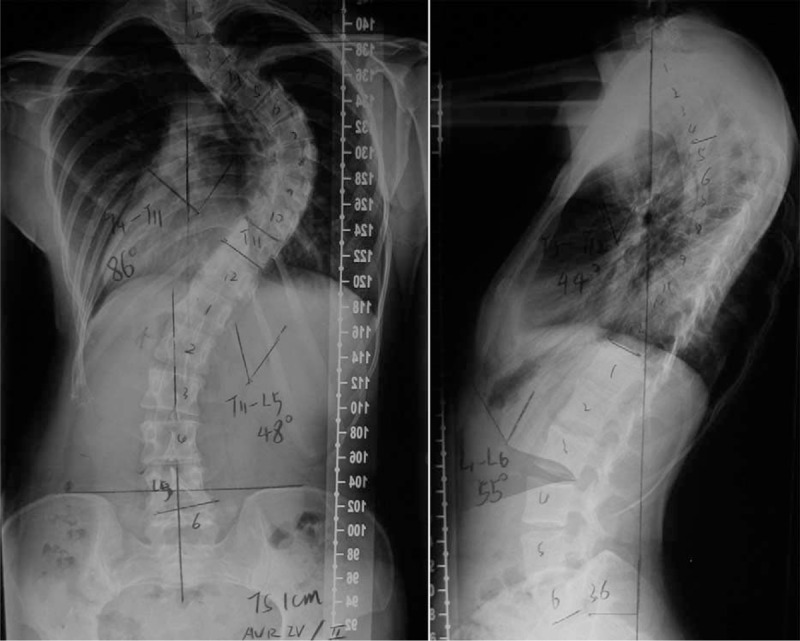
Standing anteroposterior and lateral radiographs of the preoperation.

We present this case of one 30-year-old woman who is admitted for her scoliosis deformity correction. Her radiographs with the spine demonstrated the thoracic scoliosis with Cobb angles 86° (Thoracic 4 to Thoracic 11) and thoracolumbar scoliosis with the Cobb angles 48° (Thoracic 11 to Lumbar 5) (Fig. 1), suggesting that this patient needs surgical correction.

Her past medical history was only remarkable in that she was diagnosed with hyperbilirubinaemia 2 years ago. She had undergone a liver puncture biopsy in another medical clinic that revealed the typical appearance of GS. The patient denied any recent abdominal trauma, epigastric fullness, abdominal pain, fat intolerance, and fatigue.

Magnetic resonance imaging revealed no evidence of any spinal cord or canal abnormalities. Computed tomography (CT) revealed no vertebral body deformities. Laboratory examination results were Alb 29 g/L (35–55 g/L), TBil 55.9 μmol/L (5.1 μmol/L), and DBil 4.4 μmol/L (5.1 μmol/L) (Table 1).

**TABLE 1:**
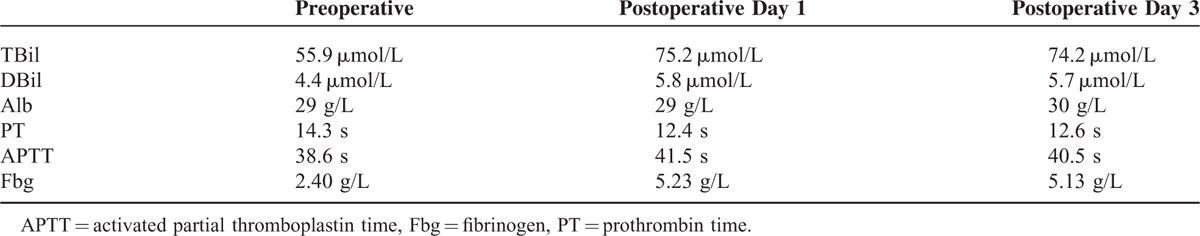
Laboratory Examination Results Between Preoperative and Postoperative

**FIGURE 2:**
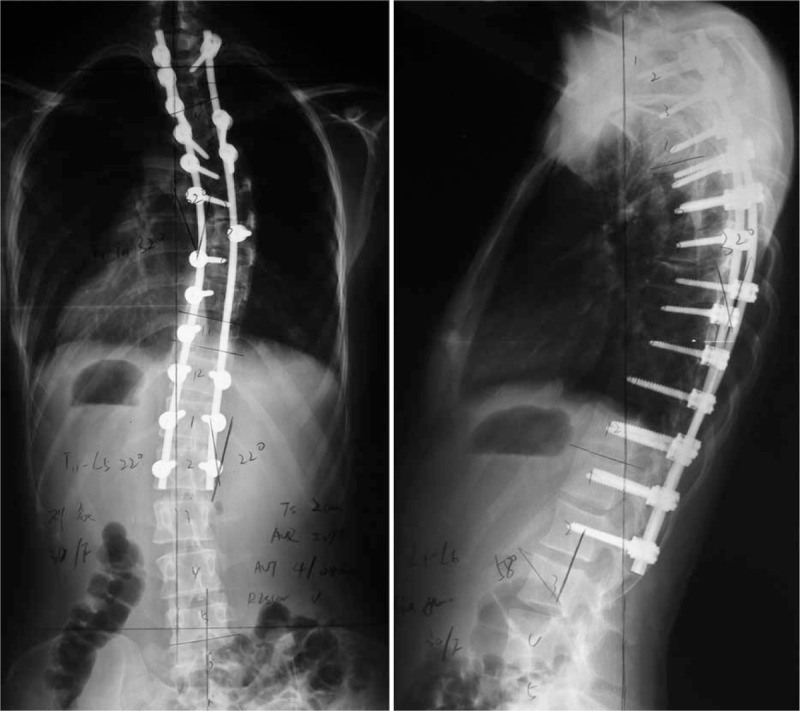
Standing anteroposterior and lateral radiographs of 4 days after operation.

**FIGURE 3:**
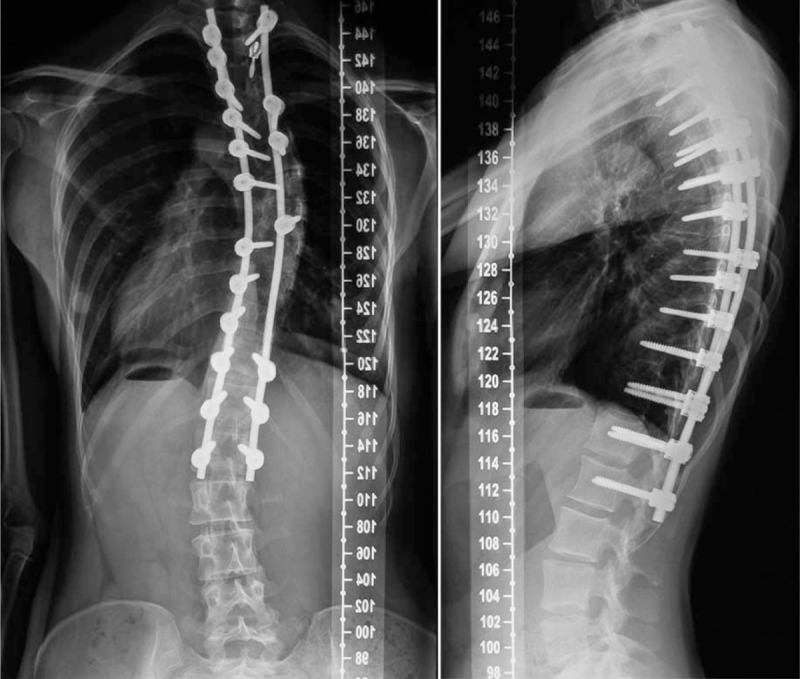
Standing anteroposterior and lateral radiographs of 12 months after operation.

In December 2012, the posterior fusion and correction at Thoracic 2 to Lumbar 1 levels were done, using a USS-II spinal system. The time of operation was 3 hours and 30 minutes. Total blood loss amount was 1500 mL, the amount of autologous blood transfusion was 1000 mL, and the amount of red blood cell (RBC) and plasma of allogenic blood transfusion was 2 μL and 200 mL, respectively. Postoperatively, it was no mild increasing of TBil with 55.9 μmol/L. Analgesic pump was used to control pain. Postoperative x-ray showed a Cobb angles correction from 86° to 32° (thoracic scoliosis, correction rate 62.8%) and 48° to 22° (thoracolumbar scoliosis, correction rate 54.2%) (Fig. 2). Her follow-up is asymptomatic, balanced in the coronal and sagittal planes, with fusion (Fig. 3) at the 24th postoperative month.

## DISCUSSION

GS is characterized by recurrent mild unconjugated hyperbilirubinaemia in the absence of haemolysis or underlying liver diseases. In GS patients, hyperbilirubinemia happens when they are exposed to operative intervention, low caloric intake, and fatigue.^1,8^ The prevalence of GS worldwide was estimated to be 7%.^10^ In this case, the diagnosis was built on the absence of overt hemolysis, mild unconjugated bilirubin increased, other liver function tests normal, and liver puncture biopsy. There are limited studies regarding the diagnosis and management of GS with scoliosis. In the present study, we reported a 30-year-old GS patient with scoliosis. To our knowledge, this is the first report of scoliosis in the setting of GS.

There are no references that describe scoliosis surgery in patients with GS. No reports are described about the safety of scoliosis surgery in GS, which need longer operation time. It is crucial to mind that preoperative hyperbilirubinemia may be a risk element for operative result.^11^ It was hard to make the best operative choice for this patient due to relative paucity of scoliosis surgical literature on GS. Many reports have evaluated surgical risks due to hepatic dysfunction such as the Child-Pugh Classification.^12^ Although the jaundice prompted by GS is slight, it is hard to see whether it will lead to liver dysfunction postoperative.

In our case, we determine to perform surgery because hyperbilirubinemia was induced by raised unconjugated bilirubin, whereas other liver function results were normal. The blood loss of our patient (1500 mL) was more than those other patients (approximately 400 mL) during the operation.^13–15^ Possible explanations for blood loss were liver dysfunction, long total operation time, and other causes. More cases are needed to study the operative risk in GS patients with scoliosis.

## CONCLUSION

Although complex scoliosis surgery could be achieved safely in GS patients, careful preoperative managements including liver function and coagulation function are required. When performing surgery on patients of scoliosis with GS, surgeons and anesthesiologists should mind the associated liver function impairment and blood loss.

## References

[1] Fretzayas A Moustaki M Liapi O Gilbert syndrome. *Eur J Pediatr* 2012; 171:11–15. 22160004 10.1007/s00431-011-1641-0

[2] Manandhar SR Gurubacharya RL Baral MR A case report of Gilbert syndrome. *Kathmandu Univ Med J (KUMJ)* 2003; 1:187–189. 16388228

[3] Lee HJ Moon HS Lee ES A case of concomitant Gilbert's syndrome and hereditary spherocytosis. *Korean J Hepatol* 2010; 16:321–324. 20924216 10.3350/kjhep.2010.16.3.321 PMC3304593

[4] Nishi H Sakaguchi T Miyagawa S Cardiac surgery in patients with Gilbert's syndrome. *J Cardiac Surg* 2012; 27:60–61. 10.1111/j.1540-8191.2011.01353.x 22150715

[5] Maruo Y Sato H Yamano T Gilbert syndrome caused by a homozygous missense mutation (Tyr486Asp) of bilirubin UDP-glucuronosyltransferase gene. *J Pediatr* 1998; 132:1045–1047. 9627603 10.1016/s0022-3476(98)70408-1

[6] Powell AJ Hansen LK Gilbert's syndrome in a patient with predominantly negative symptoms of schizophrenia. *Int J Psychiatry Clin Pract* 2007; 11:239–241. 24941364 10.1080/13651500600811735

[7] Kocer U Uysal A Sungur N Familial neurofibromatosis-1 and Gilbert syndrome. *Dermatol Surg* 2003; 29:759–765. 12828702 10.1046/j.1524-4725.2003.29192.x

[8] Radlovic N Ristic D Brdar R Association of hereditary elliptocytosis and Gilbert's syndrome as the cause of biliary calculosis: case report. *Srp Arh Celok Lek* 2011; 139:386–389. 21858981 10.2298/sarh1106386r

[9] Mohan M P LS Reddy PV Pregnancy with gilbert syndrome—a case report. *J Clin Diagn Res* 2014; 8:OD01–OD02. 10.7860/JCDR/2014/9327.4426 PMC4129268 25121033

[10] Curcio G Sciveres M Di Pisa M Refractory obstructive jaundice in a child affected with thalassodrepanocytosis: a new endoscopic approach. *BMC Gastroenterol* 2010; 10:117. 20942922 10.1186/1471-230X-10-117 PMC2964605

[11] Li Z Yu X Shen J Congential scoliosis in Wilson's disease: case report and review of the literature. *BMC Surg* 2014; 14:71. 25252715 10.1186/1471-2482-14-71 PMC4177382

[12] Kim WR Poterucha JJ Wiesner RH The relative role of the Child-Pugh classification and the Mayo natural history model in the assessment of survival in patients with primary sclerosing cholangitis. *Hepatology* 1999; 29:1643–1648. 10347102 10.1002/hep.510290607

[13] Li Z Yu X Shen J Scoliosis in Herlyn-Werner-Wunderlich syndrome: a case report and literature review. *Medicine* 2014; 93:e185. 25526433 10.1097/MD.0000000000000185 PMC4603133

[14] Li Z Shen J Liang J Thoracolumbar scoliosis in a patient with proteus syndrome: a case report and literature review. *Medicine* 2015; 94:e360. 25654373 10.1097/MD.0000000000000360 PMC4602720

[15] Li Z Shen J Liang J Scoliosis in mitochondrial myopathy: case report and review of the literature. *Medicine* 2015; 94:e513. 25674747 10.1097/MD.0000000000000513 PMC4602742

